# De Garengeot Hernia Complicated by Groin Abscess and Perforated Appendix: A Case Report and Literature Review

**DOI:** 10.1002/ccr3.73337

**Published:** 2026-08-12

**Authors:** Hadi Farhat, Khaled Al Jamal, Michel Houkayem

**Affiliations:** ^1^ Department of General Surgery, Faculty of Medicine and Medical Sciences University of Balamand El Koura Lebanon

**Keywords:** acute appendicitis, case report, De Garengeot hernia, femoral hernia, groin abscess

## Abstract

De Garengeot hernia, defined as the presence of the vermiform appendix within a femoral hernia sac, is a rare cause of acute groin pathology and often presents atypically. We report the case of a 60‐year‐old male with diabetes mellitus and chronic corticosteroid use who presented with fever and a painful, erythematous, non‐reducible right groin swelling without abdominal symptoms. Laboratory investigations showed marked leukocytosis and elevated C‐reactive protein. Computed tomography revealed a right groin abscess with intralesional air. Emergency surgical exploration demonstrated a perforated appendix protruding through the femoral canal, consistent with De Garengeot hernia. Appendectomy with drainage was performed, followed by femoral hernia repair using a McVay technique without mesh due to contamination. The postoperative course was uneventful. Early recognition and prompt surgical management are essential to achieve favorable outcomes in this rare but potentially severe condition.

## Key Clinical Message

De Garengeot hernia is a rare cause of inflammatory groin swelling and may present without abdominal symptoms. Computed tomography can aid diagnosis, but definitive recognition is often intraoperative. In contaminated cases, appendectomy with primary tissue femoral hernia repair without mesh is safe and effective.

## 1 Introduction

Femoral hernias are an uncommon subtype of groin hernias but carry a relatively high risk of incarceration and strangulation due to the narrow, rigid femoral canal [1]. De Garengeot hernia refers to the presence of the vermiform appendix within a femoral hernia sac, first described by René‐Jacques Croissant De Garengeot in 1731 [2, 3]. This entity is rare, representing a small fraction of femoral hernias, and appendicitis (or perforation) occurring within the femoral canal is even less frequent [2, 3, 4, 5]. Because the inflamed appendix is anatomically “isolated” in the femoral canal, patients often present without classic migratory right lower‐quadrant pain; instead, they typically manifest with a tender, erythematous, nonreducible groin mass that mimics an incarcerated femoral hernia, sometimes complicated by localized abscess formation [2, 4, 6].

Preoperative diagnosis remains challenging. Cross‐sectional imaging—particularly computed tomography—can demonstrate the appendix within the femoral canal and may help identify complications such as inflammation, perforation, or associated fluid/air collections, thereby assisting operative planning [2, 4, 6]. However, many cases are still diagnosed intraoperatively due to nonspecific clinical features [2, 4]. Surgical management is urgent and generally consists of appendectomy combined with femoral hernia repair; the operative approach (open vs. laparoscopic‐assisted) and the use of mesh remain debated and are frequently guided by the degree of contamination and the presence of perforation or abscess [2, 5, 7, 8]. In contaminated fields, primary tissue repair (e.g., Cooper's ligament/McVay‐type repairs) is commonly favored to reduce infectious complications [7]. We report a case of De Garengeot hernia presenting as a right groin abscess with a perforated appendix, highlighting diagnostic considerations and operative decision‐making. Consent from the patient was taken prior to writing and publishing this article.

## 2 Case History/Examination

A 60‐year‐old male patient presented with a several‐day history of fever, chills, and a painful erythematous right groin swelling. The groin bulge had progressively worsened and was associated with localized pain but no gastrointestinal symptoms. The patient denied nausea, vomiting, abdominal pain, or abdominal distension and reported normal bowel movements.

His past medical history was significant for type 2 diabetes mellitus and pulmonary fibrosis managed with chronic corticosteroid therapy. His past surgical history included bilateral inguinal hernia repair. There was no history of recent trauma.

On presentation, vital signs revealed a heart rate of 92 beats/min, blood pressure 100/70 mmHg, oxygen saturation 87% (consistent with his prior pulmonary fibrosis), and a temperature of 38.5°C. Physical examination demonstrated a tender, erythematous, warm, non‐reducible right groin mass. The abdomen was soft, non‐distended, and non‐tender, with no signs of peritonitis. The patient had initially sought medical attention with a urologist and was subsequently transferred to Batroun Hospital at approximately 11:00 PM, at which time the general surgery team was consulted. Laboratory investigations demonstrated inflammatory markers consistent with infection (Table 1).

**TABLE 1:** Initial laboratory investigations.

Parameter	Result	Unit	Reference range
White blood cells (WBC)	22,000	cells/μL	4000–11,000
Neutrophils	85	%	40–75
Hemoglobin	14	g/dL	13–17
Platelets	250,000	cells/μL	150,000–400,000
Creatinine	0.6	mg/dL	0.6–1.3
C‐reactive protein (CRP)	320	mg/L	< 5

## 3 Differential Diagnosis, Investigations and Treatment

The initial differential diagnosis included an incarcerated or strangulated femoral hernia, recurrent inguinal hernia, localized groin abscess, suppurative lymphadenitis, soft‐tissue infection, and less likely bowel‐containing hernia with perforation. Given the inflammatory nature of the groin swelling, fever, and the absence of abdominal symptoms, cross‐sectional imaging was obtained to further characterize the lesion and guide operative planning.

**FIGURE 1:**
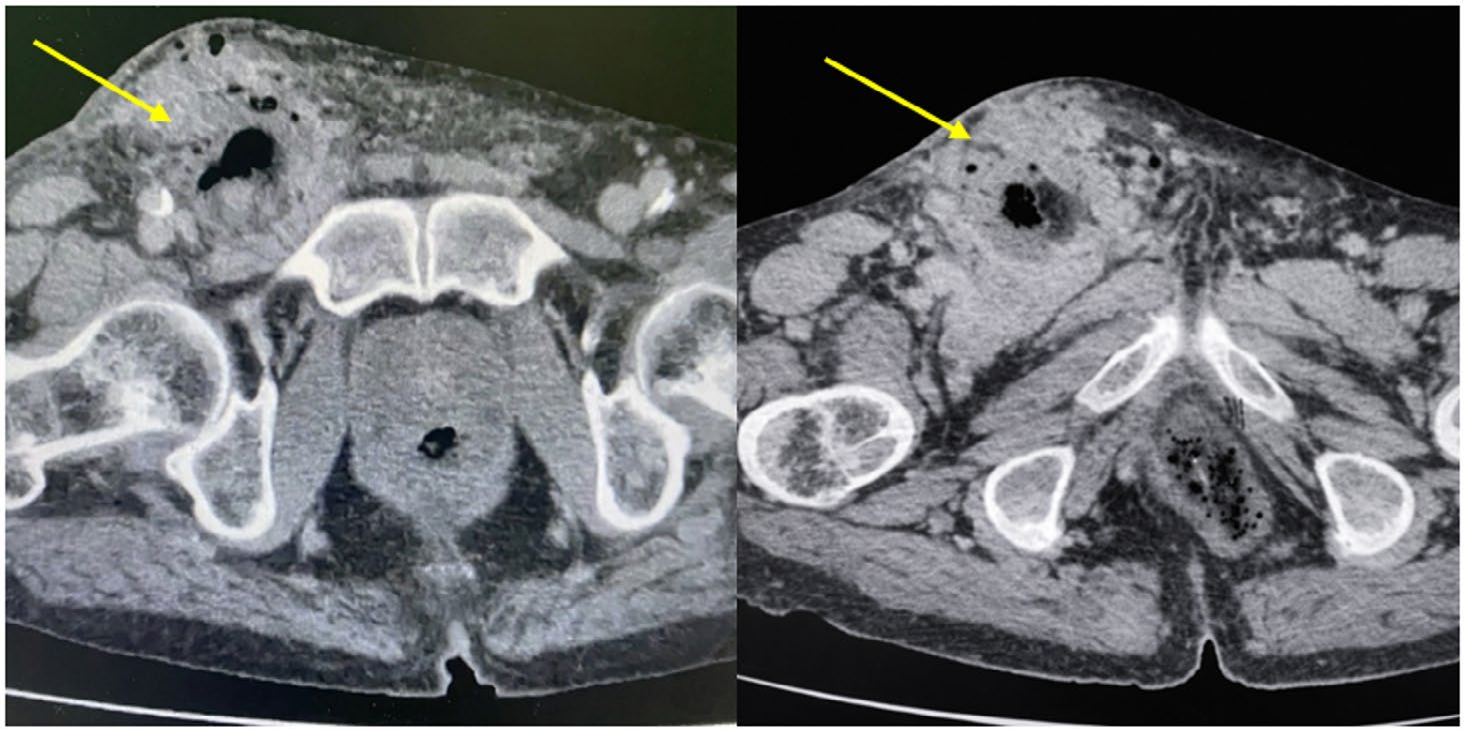
Computed tomography findings: Axial contrast‐enhanced computed tomography images of the pelvis demonstrating a right‐sided femoral hernia containing an inflamed and perforated appendix, consistent with a De Garengeot hernia. The images show a right groin fluid collection with intralesional air bubbles extending through the femoral canal, compatible with abscess formation secondary to perforated appendicitis. Surrounding fat stranding and soft tissue inflammatory changes are evident. No signs of bowel obstruction or intraperitoneal free air are observed. The left femoral canal appears unremarkable.

Given the atypical presentation and concern for a complicated groin pathology, an abdominopelvic computed tomography scan was obtained. Imaging revealed a right groin fluid collection containing multiple air bubbles, consistent with a localized abscess, without evidence of bowel obstruction or intra‐abdominal free air (Figure 1).

Given the findings, the patient was taken urgently to the operating room. A right inguinal incision was initially performed. Upon dissection, approximately 300 mL of purulent material was drained from the subcutaneous tissue beneath the fascia and ligament. Copious irrigation was performed; however, no gastrointestinal structure was immediately identified. The transversalis fascia was then opened, resulting in the evacuation of a large additional amount of purulent material.

**FIGURE 2:**
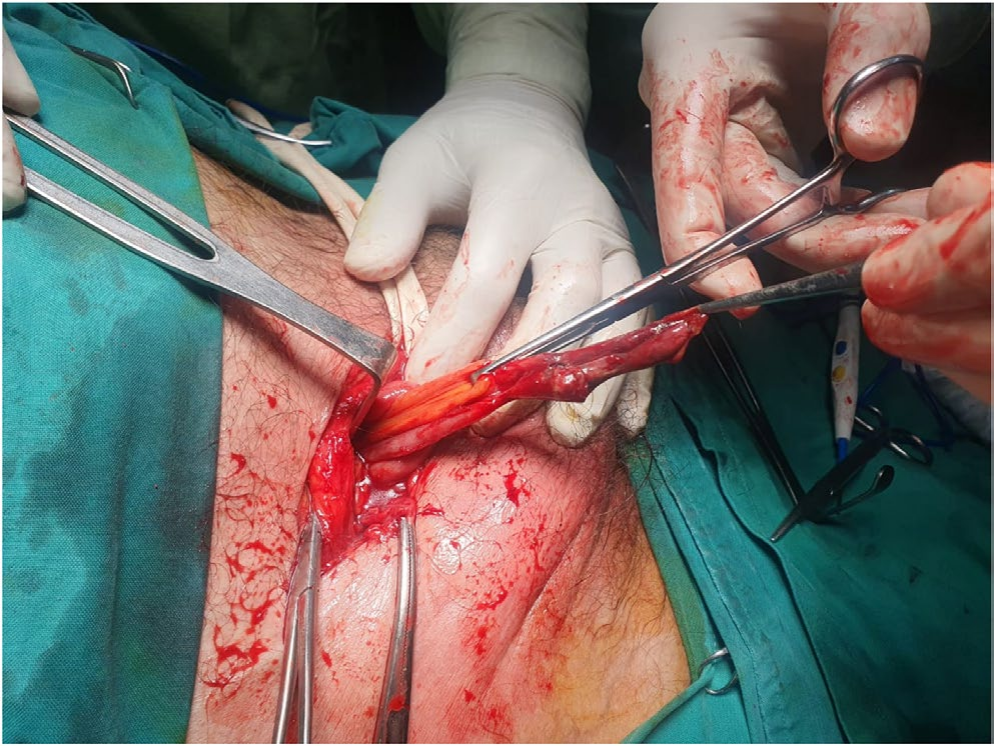
Intraoperative findings: Intraoperative photograph demonstrating extraction of the vermiform appendix through the femoral canal following blunt enlargement of the femoral hernia neck. The appendix is seen emerging from the femoral defect, confirming the diagnosis of De Garengeot hernia.

**FIGURE 3:**
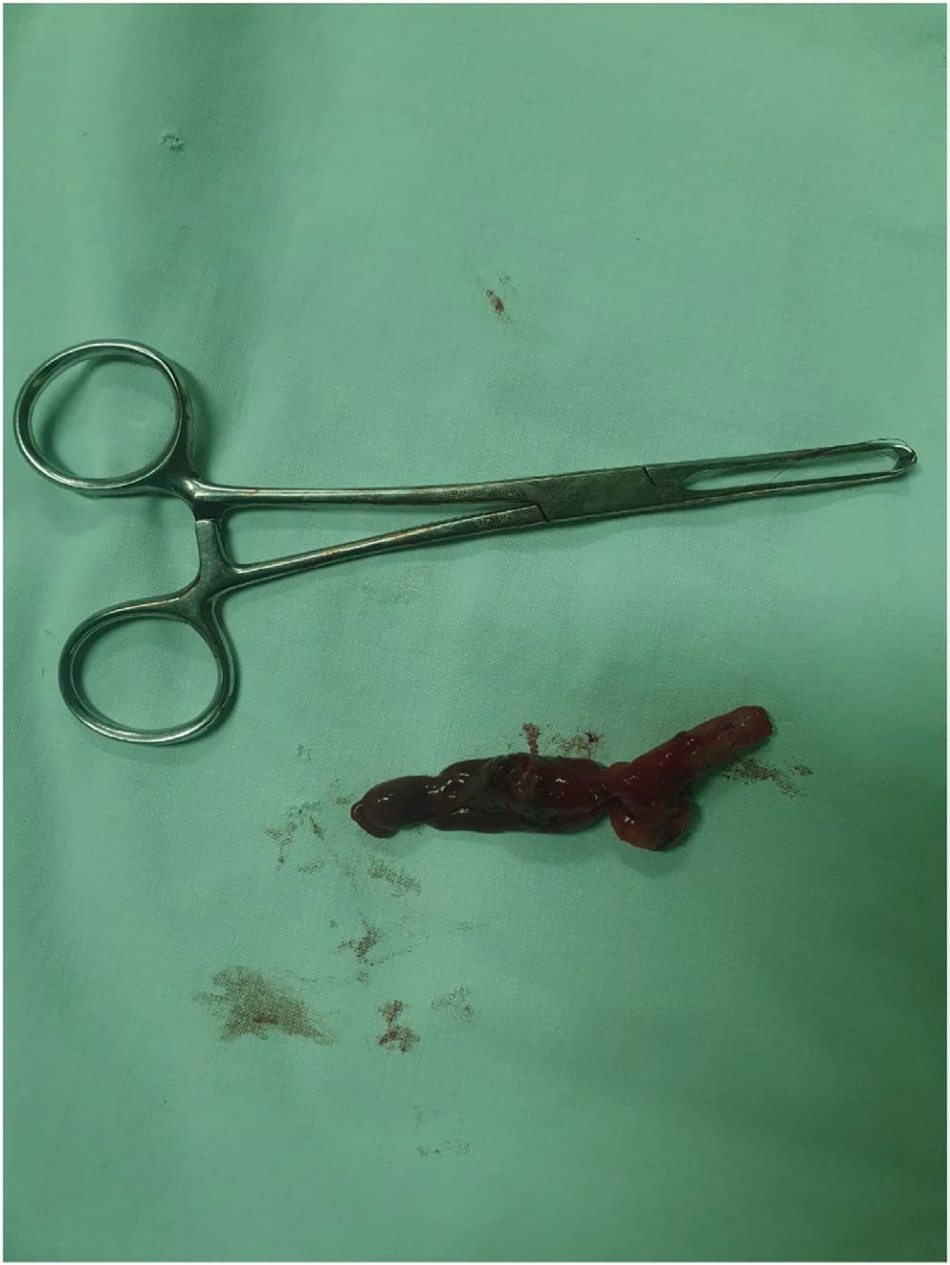
Gross specimen: Photograph of the resected appendix following appendectomy, showing macroscopic features of acute inflammation and perforation.

Following further lavage, a filiform, perforated tubular structure was visualized protruding through the femoral canal. This was identified as a perforated vermiform appendix, confirming the diagnosis of a De Garengeot hernia (Figure 2). Blunt dilatation of the femoral hernia neck was performed, allowing extraction of the appendix. The base of the appendix was identified and noted to be non‐inflamed, and a standard appendectomy was completed (Figure 3). A Penrose drain was placed in the operative field.

The femoral hernia was repaired using a McVay (Cooper's ligament) technique, with interrupted non‐absorbable sutures (Prolene 1) placed between the conjoint tendon and Cooper's ligament. No mesh was used due to the contaminated operative field.

Postoperatively, the patient was transferred to the regular surgical ward. He was started on empirical intravenous antimicrobial therapy with ceftriaxone 2 g once daily and metronidazole 500 mg every 8 h. Intraoperative purulent fluid cultures grew *Escherichia coli* , which was susceptible to the prescribed antimicrobial regimen. Intravenous antibiotics were continued for 5 days, during which the patient showed clinical improvement and a progressive decrease in inflammatory markers, with C‐reactive protein decreasing to 250 mg/L on postoperative Day 1 and to 90 mg/L on postoperative day 2.

He was then discharged on oral ciprofloxacin 500 mg every 12 h and metronidazole 500 mg every 8 h for 7 additional days, completing a total antimicrobial course of 12 days. Antibiotic therapy was initially empirical, targeting enteric Gram‐negative organisms and anaerobes in the setting of perforated appendicitis with groin abscess formation and extensive purulent contamination, and was subsequently supported by intraoperative culture results. The surgical drain was removed on postoperative day 10 during outpatient follow‐up, and the patient was noted to be clinically well with no complications.

## 4 Conclusion and Results

The patient demonstrated progressive clinical improvement after operative source control and antimicrobial therapy. His inflammatory markers decreased, with C‐reactive protein falling to 250 mg/L on postoperative Day 1 and to 90 mg/L on postoperative Day 2. After completion of inpatient intravenous antibiotics, he was discharged on oral ciprofloxacin and metronidazole to complete therapy.

The surgical drain was removed on postoperative Day 10 during outpatient follow‐up. The patient was clinically well, with no fever, wound‐related complications, recurrent groin swelling, or other early postoperative complications documented.

De Garengeot hernia is a rare and often diagnostically challenging condition that may present atypically as a painful, erythematous groin mass without classical features of acute appendicitis. This case highlights an unusual presentation complicated by perforated appendicitis and groin abscess formation, underscoring the importance of maintaining a high index of suspicion in patients with inflammatory groin swellings, particularly those with significant comorbidities or immunosuppression. Preoperative computed tomography can aid in diagnosis and surgical planning, although definitive diagnosis is frequently made intraoperatively. Prompt surgical intervention with appendectomy and femoral hernia repair remains the cornerstone of management. In contaminated operative fields, primary tissue repair without mesh is a safe and effective strategy. Early recognition and appropriate operative management are essential to achieve favorable outcomes in this rare but potentially severe clinical entity.

## 5 Discussion

De Garengeot hernia is a rare clinical entity defined by the presence of the vermiform appendix within a femoral hernia sac. Femoral hernias themselves account for less than 5% of all groin hernias and are more commonly encountered in elderly patients due to anatomical and connective tissue changes [1, 8]. The coexistence of appendicitis within a femoral hernia is exceedingly uncommon, with reported incidences ranging from 0.5% to 5% of femoral hernias, and perforation occurring in an even smaller subset of cases [1, 2, 3, 6].

The pathophysiology of appendicitis in De Garengeot hernia is thought to differ from that of typical intraperitoneal appendicitis. Rather than luminal obstruction by a fecalith, inflammation is believed to result primarily from extrinsic compression of the appendix at the narrow femoral neck, leading to ischemia and subsequent bacterial overgrowth [2, 4, 9]. This anatomical confinement also explains the atypical clinical presentation observed in most cases, including the absence of classic right lower‐quadrant abdominal pain and peritoneal signs [2, 3]. Instead, patients commonly present with a painful, erythematous, non‐reducible groin mass, often misdiagnosed as a strangulated femoral hernia or groin abscess [4, 6].

Preoperative diagnosis remains challenging. Although ultrasound may identify a groin collection, computed tomography has emerged as the imaging modality of choice, allowing visualization of the appendix within the femoral canal and identification of associated complications such as inflammation, perforation, or abscess formation [2, 4, 10, 11]. Nevertheless, despite increased use of CT imaging, many cases are still diagnosed intraoperatively due to the rarity of the condition and low preoperative suspicion [1, 6].

The present case is notable for perforated appendicitis with extensive purulent collection presenting as a groin abscess, a particularly uncommon and severe manifestation. Immunosuppression due to chronic corticosteroid use and diabetes mellitus may have contributed to delayed presentation and progression to perforation, a phenomenon also described in other reports [9, 12]. The absence of intra‐abdominal contamination and peritonitis in this patient is consistent with previous observations that the femoral canal may anatomically isolate the inflammatory process [2, 4].

Surgical management of De Garengeot hernia is universally accepted as urgent, but the optimal operative strategy remains debated. Appendectomy combined with femoral hernia repair constitutes the cornerstone of treatment [1, 2]. The surgical approach may be open, laparoscopic, or combined, depending on surgeon expertise and intraoperative findings [6, 12]. In cases complicated by perforation or abscess, as in our patient, an open approach allows effective drainage, thorough irrigation, and safe appendectomy.

The use of mesh in femoral hernia repair in the setting of contamination remains controversial. Several authors advise against mesh placement in the presence of perforation, abscess, or purulent contamination due to the increased risk of surgical site infection and mesh‐related complications [1, 7, 13]. In such circumstances, primary tissue repair techniques, including McVay (Cooper's ligament) repair, are widely recommended and have demonstrated acceptable outcomes [7, 13, 14]. In our case, mesh was deliberately avoided, and a McVay repair was successfully performed without postoperative complications.

Postoperative outcomes in De Garengeot hernia are largely dependent on the presence of perforation, delay in diagnosis, and patient comorbidities [2]. When managed promptly, prognosis is generally favorable, although reported complication rates are higher than those of routine femoral hernia repair [1, 2]. Our patient experienced an uncomplicated recovery following adequate source control, targeted antibiotic therapy, and appropriate hernia repair.

The cases most comparable to the present report are summarized in Table 2. These reports show that perforated De Garengeot hernia with groin abscess or extensive local suppuration is usually managed by open exploration, appendectomy, drainage/debridement, and non‐mesh tissue repair when contamination is present [15, 16, 17, 18, 19, 20].

**TABLE 2:** Comparison of reported perforated De Garengeot hernia cases with groin abscess or extensive local suppuration.

Author, year	Age/sex	Relevant comorbidities	Presentation	Imaging findings	Appendix/contamination	Surgical approach	Hernia repair/mesh	Outcome/follow‐up
Georgiou et al., 2013	84/M	NR	Right inguinal soft‐tissue infection	NR	Perforated appendiceal diverticulitis within a femoral hernia causing abscess and necrotizing infection of the overlying soft tissues	Surgical exploration with management of necrotizing infection	NR	NR
Bloom et al., 2017	92/F	NR	Abdominal pain followed by worsening right lower‐quadrant/groin symptoms	Initial CT showed a hernia anterior to the inguinal ligament; repeat CT showed a **7 ×** 4 cm complex right inguinal fluid collection	Appendix within the hernia sac with distal perforation	Operative exploration and appendectomy	Hernia defect repaired; mesh use NR	Tolerated procedure well; recurrence/follow‐up NR
Mashima et al., 2017	81/F	NR	Irreducible tender right groin mass	Diagnosed as incarcerated femoral hernia with right groin subcutaneous abscess	Necrotic and perforated appendix strangulated by the femoral ring with subcutaneous abscess	Inguinal incision, appendectomy through the hernia sac, lavage, and drainage	Herniorrhaphy performed; mesh use NR	Uneventful recovery; recurrence/follow‐up NR
Taveras and Huerta, 2017	94/M	Hypertension, Parkinson disease, CHF exacerbation	Acute painful right inguinal bulge; no skin changes; no leukocytosis	Abdominal series showed no obstruction	Abscess associated with femoral hernia containing the appendix	Groin exploration under local anesthesia; appendectomy through hernia sac	McVay tissue repair; no mesh reported	No complications and no recurrence at 30 days
Briceño and Jara, 2018	54/F	Obesity, hypertension, rheumatoid arthritis treated with methotrexate and prednisone	Right inguinal pain and erythema initially suspected to be cellulitis	Pelvic MRI showed perforated acute appendicitis in a groin hernia with extensive pelvic cellulitis and fasciitis	Necrotic and perforated appendix within a femoral hernia with extensive groin and pubic fasciitis	Surgical debridement, open appendectomy, vacuum‐assisted closure; required repeated debridement	Femoral hernioplasty without mesh	Discharged in good condition after 2 weeks; later wound dehiscence treated with dressings until closure
Ying and Yahng, 2019	87/F	NR	Incarcerated right femoral hernia	CT confirmed incarcerated right femoral hernia	Perforated necrotic appendix with pus contained within the hernia sac	Open hernia reduction and appendectomy	NR	NR
Present case	60/M	Type 2 diabetes mellitus, chronic corticosteroid therapy for pulmonary fibrosis	Fever, chills, painful erythematous non‐reducible right groin swelling without abdominal symptoms	CT showed a right groin fluid collection with multiple air bubbles, consistent with localized abscess, without bowel obstruction or free intra‐abdominal air	Perforated appendix protruding through the femoral canal with approximately 300 mL of purulent material and additional deep purulence	Right inguinal exploration, drainage, lavage, appendectomy, and Penrose drain placement	McVay/Cooper's ligament tissue repair with interrupted Prolene sutures; no mesh due to contamination	Uneventful recovery; discharged POD2; drain removed POD10; clinically well with no early complications

Abbreviations: CHF, congestive heart failure; CT, computed tomography; F, female; M, male; MRI, magnetic resonance imaging; NR, not reported; POD, postoperative day.

This case highlights the importance of maintaining a high index of suspicion for rare femoral hernia contents in patients presenting with atypical groin infections. Early imaging, prompt surgical intervention, and individualized operative planning remain critical to optimizing outcomes in this uncommon but potentially severe condition [2, 6].

## Author Contributions

**Hadi Farhat:** conceptualization, investigation, methodology, resources, writing – original draft, writing – review and editing. **Khaled Al Jamal:** methodology, visualization, writing – original draft, writing – review and editing. **Michel Houkayem:** investigation, methodology, project administration, supervision, writing – review and editing.

## Funding

The authors have nothing to report.

## Ethics Statement

This study was conducted in accordance with the principles of the Declaration of Helsinki. Ethical approval was waived by the Institutional Review Board of Batroun Hospital, Batroun, Lebanon, as this work represents a single‐patient case report with no experimental intervention and no deviation from standard clinical care. Approval number was not applicable.

## Consent

Written informed consent for publication of the patient's clinical data and accompanying images was obtained from the patient. Non‐essential identifying details have been omitted to preserve patient confidentiality. The signed consent form is retained by the authors and is available upon reasonable request.

## Conflicts of Interest

The authors declare no conflicts of interest.

## Data Availability

The authors have nothing to report.
